# *In Silico* Analysis of Missense Mutations in *LPAR6* Reveals Abnormal Phospholipid Signaling Pathway Leading to Hypotrichosis

**DOI:** 10.1371/journal.pone.0104756

**Published:** 2014-08-13

**Authors:** Syed Irfan Raza, Dost Muhammad, Abid Jan, Raja Hussain Ali, Mubashir Hassan, Wasim Ahmad, Sajid Rashid

**Affiliations:** 1 Department of Biochemistry, Faculty of Biological Sciences, Quaid-i-Azam University, Islamabad, Pakistan; 2 National Center for Bioinformatics, Faculty of Biological Sciences, Quaid-i-Azam University, Islamabad, Pakistan; 3 Army Medical College, National University of Science and Technology NUST, Islamabad, Pakistan; A*STAR, Singapore

## Abstract

Autosomal recessive hypotrichosis is a rare genetic irreversible hair loss disorder characterized by sparse scalp hair, sparse to absent eyebrows and eyelashes, and sparse axillary and body hair. The study, presented here, established genetic linkage in four families showing similar phenotypes to lysophosphatidic acid receptor 6 (*LPAR6*) gene on chromosome 13q14.11-q21.32. Subsequently, sequence analysis of the gene revealed two previously reported missense mutations including p.D63V in affected members of one and p.I188F in three other families. Molecular modeling and docking analysis was performed to investigate binding of a ligand oleoyl-L-alpha-lysophosphatidic acid (LPA) to modeled protein structures of normal and mutated (D63V, G146R, I188F, N248Y, S3T, L277P) LPAR6 receptors. The mutant receptors showed a complete shift in orientation of LPA at the binding site. In addition, hydropathy analysis revealed a significant change in the membrane spanning topology of LPAR6 helical segments. The present study further substantiated involvement of LPAR6-LPA signaling in the pathogenesis of hypotrichosis/woolly hair and provided additional insight into the molecular mechanism of hair development.

## Introduction

Autosomal recessive hypotrichosis is a rare form of alopecia characterized by sparse hair on scalp, sparse to absent eyebrows and eyelashes, and sparse auxiliary and body hair. Over the past few years, mutations in at least eight genes causing variable phenotypes of autosomal recessive form of hypotrichosis have been reported. Except in two cases [1], [2], the underlying disease causing genes in rest of the six forms of hypotrichosis have been reported. Mutations in two of these genes, *LPAR6* and *LIPH*, result in similar features including hypotrichosis and woolly hair [3]. Presence of woolly hair has also been reported in patients carrying mutations in keratin-74 gene with phenotype segregating in autosomal dominant fashion [4].

The *LPAR6* is a nested gene residing within intron-17 of the *RB1* and encodes a heptaspan transmembrane protein that belongs to an orphan G-protein-coupled receptor (GPCR). Both LIPH and LPAR6 express in the inner root sheath (IRS) of hair follicle [5], [6], [7] and are involved in the same pathway of hair growth regulation and differentiation [8]. Oleoyl-Lalpha-lysophosphatidic acid (LPA), a bioactive lipid and product of LIPH serves as a ligand for LPAR6 receptor [8].

In the present study, screening *LPAR6* in four consanguineous Pakistani families led to the identification of two previously reported missense mutations (p.D63V, p.I188F). Modeling and mutagenesis studies of six missense mutations (Table 1), including two identified here, in *LPAR6* gene were performed to delineate their significance at respective residual level. In addition, binding of mutated LPAR6 with LPA was assessed to map the conformational changes in ligand binding pocket.

**Table 1:** Missense mutations and the resultant phenotypes observed in the families described in the present study.

S. No.	Phenotype	cDNA change	Substitution	Reference
1	Wooly hair	c.188A>T	D63V	In family A [3], [6], [21]
2	Hypotrichosis/Woolly hair	c.562A>T	I188F	In family B, C, D [3], [6]
3	Hypotrichosis	c.436G>A	G146R	[3], [22]
4	Hypotrichosis	c.8G >C	S3T	[22]
5	Hypotrichosis/Woolly hair	c.565G>A	E189K	[6], [22]
6	Hypotrichosis	c.742A>T	D248Y	[25]

## Materials and Methods

### Human subjects

**Figure 1:**
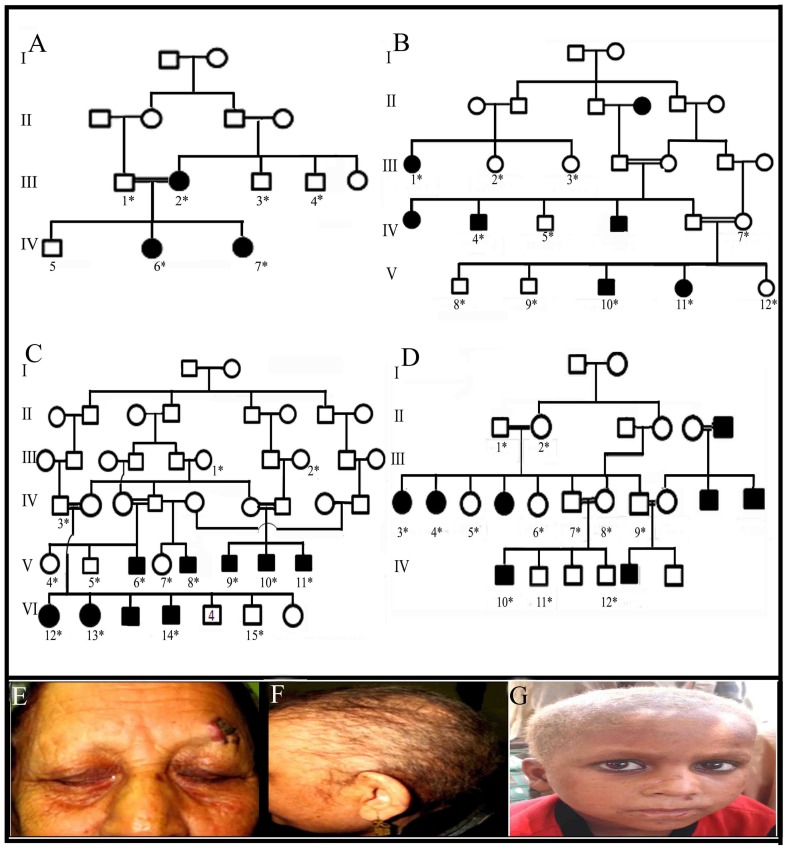
Pedigrees of four families. (A–D) Each of the four families segregated hereditary hypotrichosis and showed linkage to the *LPAR6* gene. Circles and squares represent females and males, respectively. Clear symbols represent unaffected individuals while filled symbols represent affected individuals. Double lines are indicative of consanguineous unions. (E–G) Affected members III-2 and IV-6 (family A) and IV-13 (family C) showed woolly scalp hair/sparse scalp hair, sparse eyebrows and eyelashes.

The study, presented here, describes clinical and molecular analyses of four consanguineous families (A-D) segregating autosomal recessive form of hypotrichosis/woolly hair phenotype. The study was approved by the Institutional Review Board (IRB) of Quaid-i-Azam University, Islamabad, Pakistan. Informed consent was obtained from all those participated in the study. In total, there were 27 affected and 83 unaffected individuals in the 4 families investigated in the present study (Figure 1).

### Extraction of genomic DNA and genotyping

Venous blood samples from both affected and unaffected members in each of the four families were collected in EDTA containing vacutainer sets. DNA was extracted from the available blood samples using GenElute Blood Genomic DNA Kit (Sigma-Aldrich, MO, USA). Quantification of genomic DNA was carried out by measuring optical density at 260 nm and diluted to 40–50 ng/µl for amplification by polymerase chain reaction (PCR).

### Genetic analysis

Based on the clinical features observed in affected individuals, all the four families were tested for linkage using microsatellite markers mapped to the chromosomal regions harboring two genes (*LIPH*, *LPAR6*) involved in autosomal recessive hypotrichosis. The microsatellite markers used to test linkage in the families included D3S2314, D3S1618, D3S3609, D3S3583, D3S3592 and D3S1530 linked to *LIPH* gene on chromosome 3q27.2; and D13S1312, D13S168, D13S164, D13S273, D13S284 and D13S1807 linked to *LPAR6* on chromosome 13q14.11-q21.323. The amplification of microsatellite markers was performed according to standard procedure in a total volume of 25 µl. PCR was carried out in a 0.2 ml PCR tube with 40 ng human genomic DNA, 2.5 µl reaction buffer (KCl 50 mM, Tris-Cl pH 8.3), 15–20 pmol of each primer, 1.5 mM MgCl_2_, 200 µM of each deoxynucleoside triphosphate (dNTP), and 1 unit of Taq DNA polymerase (MBI Fermentas, Life Sciences, UK). The standard thermal conditions were used as described elsewhere [6]. The PCR was performed in T3000 thermocycler (Biometra, Göettingen, Germany) and PCR products were resolved on 8% non-denaturing polyacrylamide gel. Gels were stained with ethidium bromide and genotypes were assigned by visual inspection.

The entire 1032 bp coding sequence of *LPAR6* gene was sequenced in all available affected and unaffected members of the four families. Purification of the PCR-amplified product was performed with commercially available kits (Marligen Biosciences, Ijamsville, MD, USA). Bidirectional DNA sequencing was performed using DTCS Quick Start sequencing kit (Beckman Coulter, Fullerton, CA, USA) according to the manufacturer's instructions. Sequence variants were identified via BIOEDIT sequence alignment editor version 6.0.7.

### 3D structure prediction

In the absence of a well-defined or experimentally determined structure, comparative modeling is one of the most accurate computational approaches to generate a reliable tertiary protein structure through sequence information [9]. Primary protein sequence for LPAR6 (ID: ENST00000345941) was retrieved through Ensembl Genome Browser (http://www.ensembl.org) and subjected to BLAST search against protein data bank (http://www.rcsb.org) for suitable template search. Three dimensional (3D) structure of LPAR6^WT^ was predicted through MODELLER 9V8 tool [10] using crystal structure of human kappa opoid receptor (PDB ID: 4DJH) as template (resolution: 2.90 Å). The structures of mutated LPAR6 (D63V, G146R, I188F, N248Y, S3T and L277P) were predicted through respective residue function using MODELLER 9V8 tool. Subsequently, Ramachandran plot, PROCHECK [11], ERRAT [12], VERIFY 3D [13] and WHATIF [14] tools were utilized to validate these models, followed by model refinement, editing and geometry optimization using UCSF Chimera 1.7.0 [15] and VEGA ZZ [16] tools.

### Hydropathy analysis

In order to assess and distinguish the transmembrane (TM) helices of normal and mutated LPAR6 (D63V, G146R, I188F, N248Y, L277P), hydrophobicity analyses of respective amino acids were performed using Membrane Protein Explorer (MPEx) [17] and TransMembrane protein Re-Presentation in 2 Dimensions (TMRPres2D) tools [18]. MPEx tool uses experimental values of hydrophobicity scales based on physical and biological analyses to predict the TM regions. It is widely used to evaluate the effect of a particular mutation in TM helices. TMRPres2D tool is used for 2D visualization of TM segments.

### Molecular docking analysis

Molecular docking assays for modeled LPAR6^WT^ and LPA interaction were carried out using AutoDock 4.0 tool [19] on OpenSUSE 11.2 containing Intel(R) Core (TM) i5-2300 CPU system. The structure of LPAR6 ligand designated as LPA was retrieved through PDB database (PDB ID: NKP) and optimized by performing energy minimization steps using MMFF94 force field. Docking analysis was performed as described previously [20]. Briefly, polar hydrogen atoms were added and Kollman charges were assigned for ligand and all torsions were set free to rotate in order to perform docking experiments with a rigid receptor and flexible ligand. Annealing parameters for hydrogen bonding and Van der Waals interactions were set to 4.0 Å and 2.5 Å. A grid map of 80×80×80 points with a spacing of 0.875 Å was set on the whole protein structure to generate the grid map. The number of runs for each docking experiment was set to 100. The empirical free energy function and Lamarckian genetic algorithm (LGA) were applied with the following parameters: population of 150 randomly placed individuals, a maximum number of 27,000 generations, a mutation rate of 0.02, a crossover rate of 0.80 and number of energy evaluations was 2.5×10^6^, the remaining docking parameters were set to default. The program automatically grouped potential receptor-ligand complex conformations into clusters based on their RMSD, using the default threshold (2.0 Å RMSD). The best docked complex for LPAR6^WT^-LPA was selected on the basis of binding free energy value and interactions were monitored using UCSF Chimera 1.7.0 tool [21].

## Results

### Clinical findings

Affected members of the four families (A–D) showed typical features of hereditary hypotrichosis including fragile scalp hairs, sparse to absent eyebrows and eyelashes, and sparse body hairs (Figure 1). Males and females were equally affected. Other ectodermal structures such as nails, teeth and sweat glands were normal in the affected members. Obligate heterozygous carrier individuals in each family had normal scalp and body hairs and were clinically indistinguishable from genotypically normal individuals.

### Genotyping and mutation analysis

Based on clinical features exhibited by affected individuals, all the four families were tested for linkage to genes *LIPH* and *LPAR6* mapped on chromosome 3q and 13q14.11-q21.32, respectively. Genotyping data showed linkage of all the 4 families to the *LPAR6* gene. Subsequently, *LPAR6* was sequenced in two affected and one unaffected individual of each of the four families. Sequence analysis of the *LPAR6* was performed using a control reference sequence obtained from the Ensembl database (Ensembl accession ID: ENSG00000139679).

**Figure 2:**
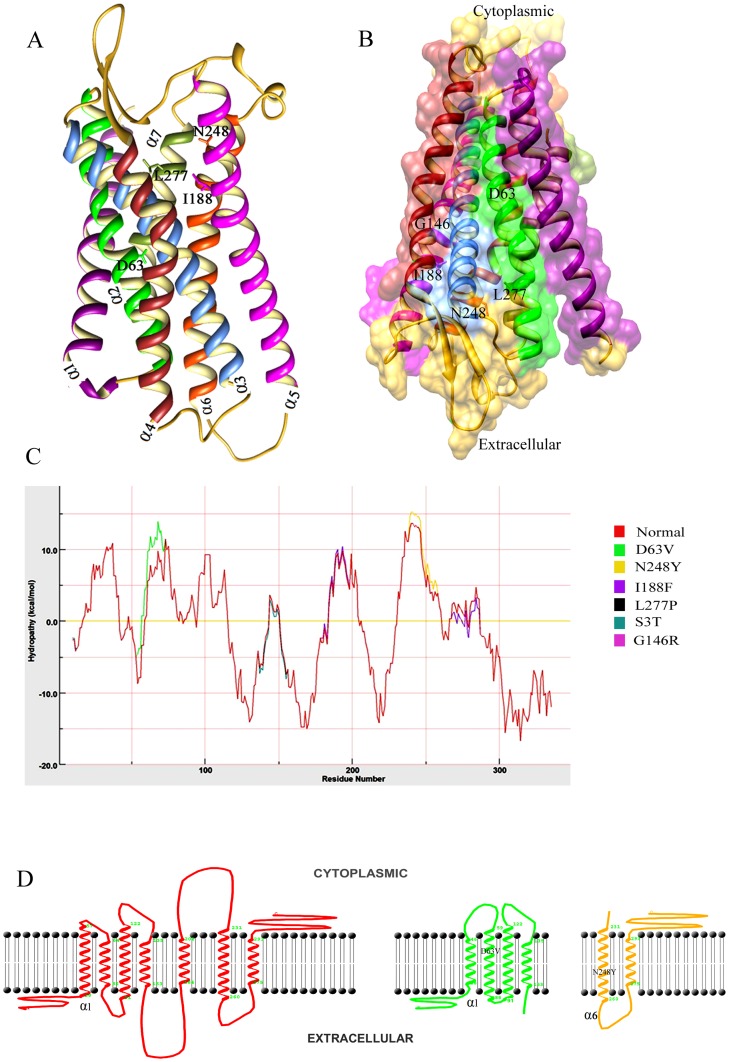
Characterization of LPAR6 specific mutations at structure level. (A) 3D representation of LPAR6structure in ribbon form. Individual α-helices are indicated by distinct colors: α1, magenta; α2, green; α3, cornflower blue; α4, brown; α5, pink; α6, orange red and α7, olive. β-sheets are indicated by yellow color. (B) Schematic representation of LPAR6 secondary structures indicating the directionality of β-sheets to the extracellular part, while narrow end of α-helices points to the cytosol, making a groove-like structure. Corresponding positions of known amino acids undergoing substitutions are indicated which show that I188, N248 and L277 lie to the extracellular part of α-helices, while D63 and G146 residues are near the cytosolic part. Surface view of groove-like structure is shown to visualize the residual positions. (C) Hydropathy plot analysis of normal and mutated LPAR6 amino acids performed by MPEx tool. (D) Membrane spanning profile studies of individual α-helices for LPAR6^WT^, LPAR6^D63V^ and LPAR6^N248Y^.

Mutation analysis of the *LPAR6* in affected individuals of the four families revealed two previously described mutations. In family A, sequence analysis revealed a previously reported missense mutation involving transition of A>T at nucleotide position 188 (c.188A>T) [6], [22]. This substitution resulted in replacing the negatively charged aspartic acid residue with a nonpolar aliphatic valine at position 63 (p.D63V) of LPAR6. Sequence analysis of the *LPAR6* in the affected members of other three families (B, C, D) revealed the other reported missense mutation involving substitution of Isoleucine with Phenylalanine at amino acid position 188 (c.562A>T, p.I188F) (Figure 2) [3].

### Hydropathy pattern analysis

**Figure 3:**
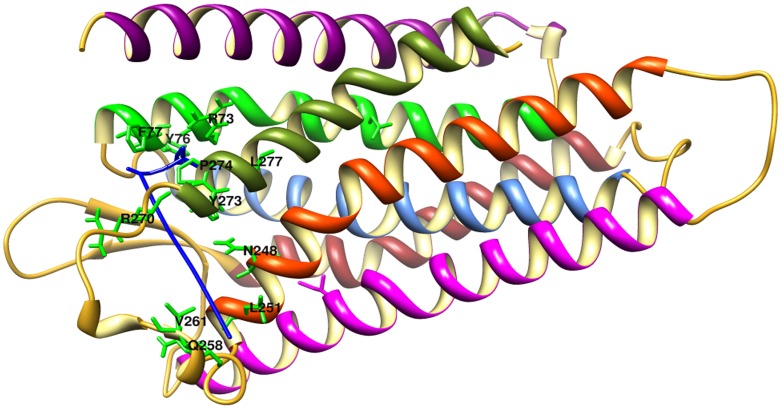
Residual contributions explored through molecular dockings of LPAR6^WT^ and LPA binding. Labels are indicated by black color. Individual α-helices are indicated by distinct colors: α1, magenta; α2, green; α3, cornflower blue; α4, brown; α5, pink; α6, orange red and α7, olive. Loop regions and β-sheets are indicated by gold color.

To evaluate the functional impact of known mutations (D63V, G146R, I188F, N248Y and L277P) in LPAR6, we first characterized their structural placements. As shown in Figure 3A, I188, N248 and L277 lie towards the extracellular part of helices, while D63 and G146 residues are localized towards the cytosolic part (Figures 2A and B).

In order to evaluate transmembrane helices for normal and mutated LPAR6, hydrophobicity analysis of individual amino acids was performed and helical conformations spanning the membrane were carefully analyzed. By monitoring the favorable protein transmembrane regions through hydropathy plot (Figure 2C), D63V and N248Y substitutions were found significant and were chosen for detailed analysis. Evidently, placement of helical segments in LPAR6^D63V^ were modified, compared to LPAR6^WT^ (Table 2), resulting in intracellular shifting of α-helix1 and pushing the onward extracellular part to the membrane side (Figure 2D). In case of LPAR6^N248Y^, we observed a slight increase of ΔG score for α-helix6 (Table 2), compared to LPAR6^WT^. However, there was no significant change in the topology of membrane segments (Figure 2D). These results clearly demonstrated that D63V substitution exerted more severe effect in membrane spanning of LPAR6 helices.

**Table 2:** Hydropathy analysis results for LPAR6^WT^, LPAR6^D63V^ and LPAR6^N248Y^ proteins.

LPAR6	Hydropathy Segment	Position	ΔG score
Normal	FVLGLISNCVAIYIFICVLKVRNETTTYMI	28–57 (30)	3.94
	LLFVFTLPFRIFYFTTRNW	64–82 (19)	11.32
	ISVMLFYTNMYGSILFLTCISVDRFLAIVYPF	91–122 (32)	11.22
	IVCTGVWLTVIGGSAPAVF	135–153 (19)	3.6
	IVIFIEIVGFFIPLILNVT	184–202 (19)	9.71
	IFVhLIIFCFCFVPYNINLILYSLVRTQTF	231–260 (30)	16.5
	ITLCIAVSNCCFDPIVYYF	275–293 (19)	4.59
D63V	FVLGLISNCVAIYIFICVL	28–46 (19)	10.82
	LAMSVLLFVFTLPFRIFYFTTRNWPFGDLL	59–88 (30)	12.02
	ISVMLFYTNMYGSILFLTCISVDRFLAIVYPF	91–122 (32)	11.22
	IVCTGVWLTVIGGSAPAVF	135–153 (19)	3.6
	IVIFIEIVGFFIPLILNVT	184–202 (19)	9.71
	IFVhLIIFCFCFVPYNINLILYSLVRTQTF	231–260 (30)	16.5
	ITLCIAVSNCCFDPIVYYF	275–293 (19)	4.59
N248Y	FVLGLISNCVAIYIFICVLKVRNETTTYMI	28–57 (30)	3.94
	LLFVFTLPFRIFYFTTRNW	64–82 (19)	11.32
	ISVMLFYTNMYGSILFLTCISVDRFLAIVYPF	91–122 (32)	11.22
	IVCTGVWLTVIGGSAPAVF	135–153 (19)	3.6
	IVIFIEIVGFFIPLILNVT	184–202 (19)	9.71
	IFVhLIIFCFCFVPYNIYLILYSLVRTQTF	231–260 (30)	18.06
	ITLCIAVSNCCFDPIVYYF	275–293 (19)	4.59

### Binding orientation and interaction mode studies

In order to assess the conformational changes produced in LPAR6 due to its interaction to ligand LPA, a predicted 3D structure of LPAR6^WT^ (D13-W307 residues) using human kappa opoid receptor (PDB ID: 4DJH) as template (27% sequence identity) was investigated. Further, the predicted structure was refined and characterized using structure analysis tools available online. Ramachandran plot for the predicted LPAR6^WT^ model indicated the presence of approximately 97.37% residues in favorable region. In addition, other factors including peptide bond planarity, non-bonded interactions, Cα tetrahedral distortion, main chain H-bond energy, poor rotamers and overall G-factor for the modeled structures lied within the favorable range. The mutated LPAR6 structures were modeled using LPAR6^WT^ structure.

### Docking analysis of LPAR6 and LPA

**Figure 4:**
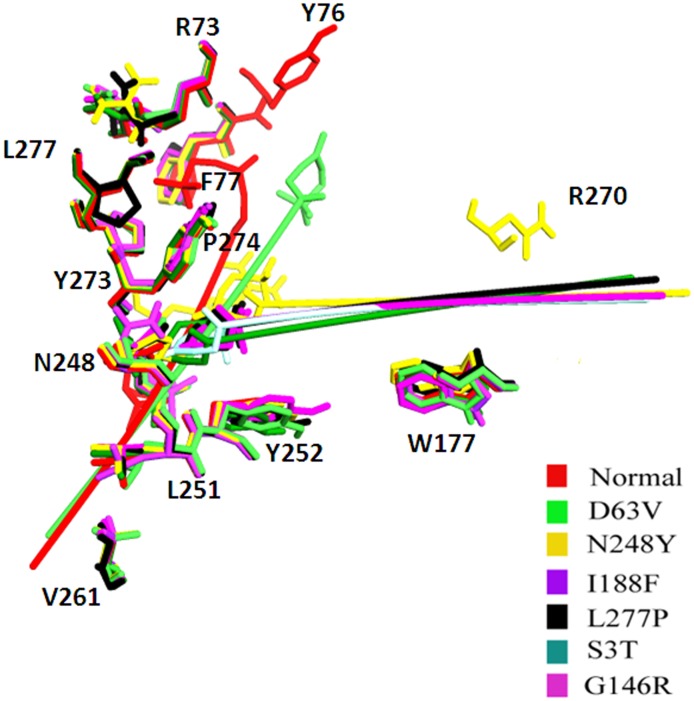
Comparative analysis of conformational changes in LPA-bound LPAR6 structures at residual level. Individual mutations are indicated by distinct colors. Binding residues are labeled in black color.

Through docking simulations, more pronounced changes were observed in the β-sheets (N155-S159 and E166-E170), located at the extracellular part of LPAR6. In LPAR6^WT^-LPA complex, R73, Y76, F77, N248, L251, Q258, V261, C263, R270, Y273, P274, L277 and C278 residues of LPAR6 were involved in LPA binding (Figure 3). Interestingly, two residues N248 and L277, found mutated (N248Y and L277P) in families with hypotrichosis/woolly hair, were actively involved in binding LPA (Figure 4). Overall, total binding energy for LPAR6^WT^-LPA interaction was -7.94 Kcal/mol. A comparative binding analysis of interaction of LPA with LPAR6^WT^ and LPAR6^MT^ proteins are described in Table 3 and Figure S1.

**Table 3:** Comparative binding analysis of LPAR6^WT^ and LPAR6^MT^ structures bound to LPA. Similar residues in multiple complexes are indicated in bold.

Structure	RMSD (Å)	Binding energy (Kcal/mol)	Interacting Residues
**LPAR6^WT^**	0	−7.94	Y76, P77, R73, P274, **Y273, N248, Y252, V269**, L251, L277, **V261**
**LPAR6^D63V^**	0.447	−7.78	R80, N81, W82, E166, **Y273**, M272, **V269, N248, V261**
**LPAR6^G146R^**	0.379	−7.87	**Y273**, F169, **V269, N248**, N171, F172, **Y252**, L181, **W177**, E174
**LPAR6^I188F^**	0.379	−6.24	R270, **Y273**, M272, **V269**, F169, N171, **N248**, L249, F172, L181, **Y252, W177**, E174
**LPAR6^L277P^**	0.253	−8.28	R270, **Y273**, F169, **N248**, N171, **Y252**, F172, L181, **W177**, E174
**LPAR6^N248Y^**	0.274	−8.4	R270, **Y273**, F169, N171, **Y248**, F172, **W177**, E174
**LPAR6^S3T^**	0.199	−8.24	R270, V269, **Y273**, F169, **N248**, N171, **Y252**, F172, L181, **W177**

## Discussion

To date, several mutations in the hair-follicle specific epithelial keratins have been reported which are associated with exhibition of curly-wavy phenotype [23], woolly hair and hypotrichosis in humans [4], [24]. Notably, clinical features observed in the affected members of these families were similar to those reported in the patients carrying mutations in either *LPAR6* or *LIPH* gene (Table 1). It has been recently shown that epithelial keratins colocalize with LPAR6 in the Henle's (He) and Huxley's (Hux) layers of follicle-specific inner root sheath (IRS) [4]. Consequently, hair follicle development requires an accurate binding of LPA (a product of LIPH) to LPAR6 receptor for the subsequent keratinization process, leading to the formation of hair shaft. The missense mutations [9], [25], [26], [27] and deletions [7], [8], [28] in *LPAR6* may disturb the underlying downstream signaling events occurring in the inner root sheath (IRS) hair follicle.

**Figure 5:**
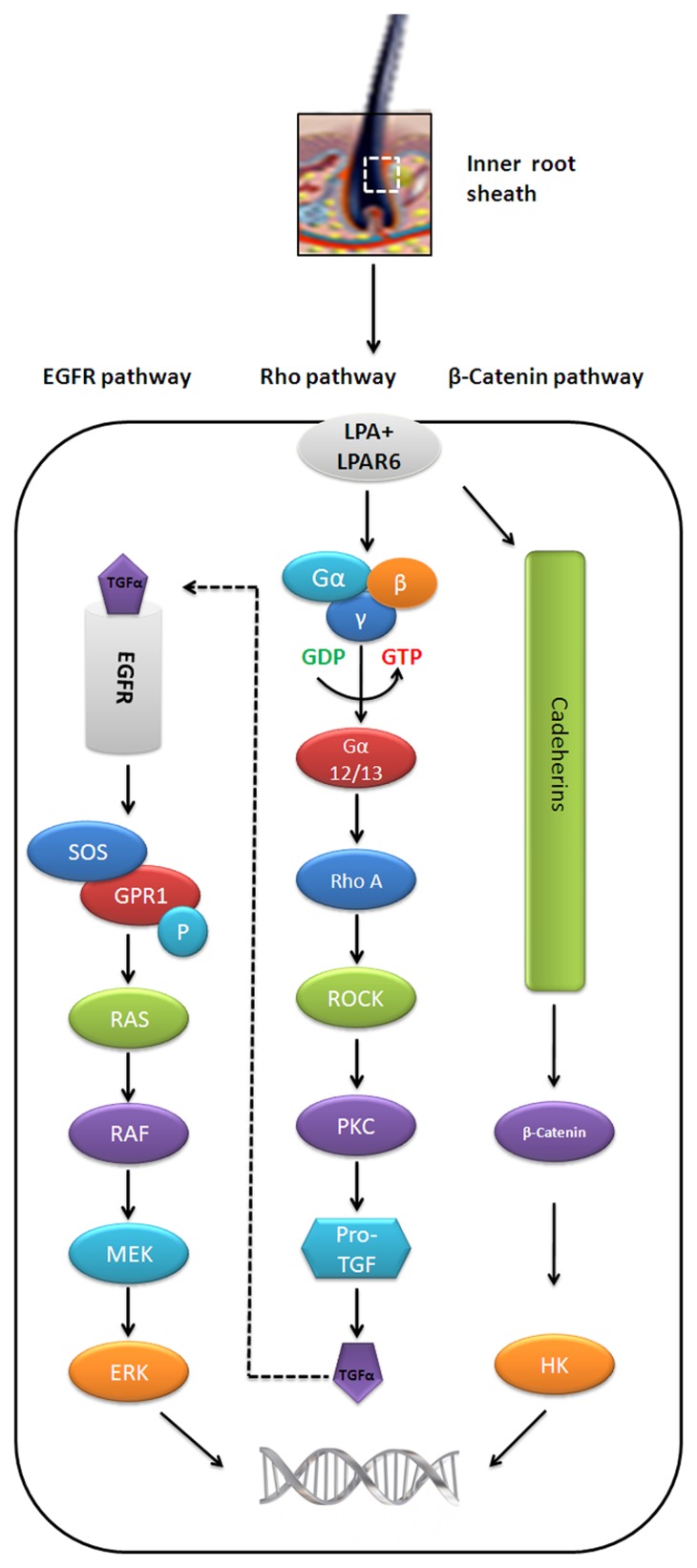
Overview of LPAR6-LPA dependent pathways involved in hair follicle development. Three possible pathways (β-Catenin, Rho and EGFR) are known to occur in the inner root of hair sheath. A dotted line arrow indicates TGF-α inter-linkage with EGFR pathway. The β-catenin together with Cadherins may activate HK genes and help in hair development [34]. Rho and EGFR pathways are linked with each other by functional activity of TGF-α which is directly involved in the activation of EGFR. Rho signaling cascade (GTP binding proteins) and EGFR proteins activate MEK/ERK which results in the development of hair follicle by regulating the downstream transcriptional activity [35], [36]. HK, hair keratins; G-Protein αβδ, GTP binding proteins; Gα-12/13, Subunit of GTP binding protein alpha; RhoA, Ras homolog gene family member A; ROCK, Rho associated protein kinase; PKC, Protein kinase C; Pro TGF, Protein transforming growth factor; EGFR, Epidermal growth factor receptor; SOS protein, son of seveless protein; GPR1, G-protein couple receptor 1; MEK, MAPK/ERK kinases; ERK, extracellular signal regulated kinases.

A closer association between hair growth abnormalities and spectrum of mutations reported in *LPAR6* demands a detailed characterization of underlying signaling events. Generally, LIPH encoding enzyme, Lipase H is involved in the formation of LPA from phosphatidic acid which elicits the signal transduction mechanism (Figure 5) by binding to purine and pyrimidine nucleotide receptor LPAR6 [7]. In this study, through 3D structure prediction of LPAR6 and localization of missense mutations followed by elucidation of LPA binding pattern to normal and mutated LPAR6, we evaluated the residual contributions in mediating downstream signaling events.

Despite sharing a common binding region (Table 3), there exist significant conformational transitions in the LPA binding pattern to normal and mutated forms of the receptor. The four missense mutations (S3T, G146R, I188F, L277P) completely shifted the orientation of LPA at binding site of the receptor. These substitutions altered the affinity of LPAR6 receptor to LPA ligand thereby disturbing the downstream signaling pathway, which governs wooly hair formation in human. However, except D63V, other mutations failed to alter the topology of transmembrane helices. These findings largely underscore that regulation of lipid metabolism resulting from coupling of LPA to its receptor is a key factor in hair development and differentiation.

The comparative binding analyses of LPAR6 indicated the predominant involvements of F172, W177, L181, N248, Y252, V261, V269, R270 and Y273 residues in LPA interactions to its receptor. Notably, N248 and L277 residues were frequently observed in the receptor-mediated interactions, indicating N248Y and L277P substitutions may have more severe effect in development of phenotype. These findings are in good agreement with the features of less dense and/or sparse hair observed in families carrying such mutations (Table 1).

In binding of LPA to LPAR6 two aliphatic amino acids, V and L, preferably localize in the middle of lipid bilayer, while two other residues, W and Y, facing towards the membrane surface are involved in contacting membrane spanning α-helices with long chain fatty acid of LPA. Clearly, LPA acts as a multifunctional signaling molecule due to incorporation of free hydroxyl, phosphate moiety and hydrophilic nature [29]. However, it is important to address any change occur in biological activity of LPA as a result of transition of its conformational features, particularly those related to binding to mutated LPAR6. Indeed, LPA activity is dependent on the length of its acyl chains (C_16_ to C_20_) and decreases by the shortening of chain length [30]. It has been postulated that phosphate group of LPA is pivotal for its activity and the acyl chains tend to anchor it in the hydrophobic cavity for the optimal orientation of phosphate group to interact with positively charged residues of receptor [30]. However, in LPAR6-LPA model, we detected the participation of neutral amino acids (F77, P274, L277) in phosphate group binding. A somewhat different picture resulted for the mutated LPAR6 with respect to orientation of phosphate group. It seems more likely that the mutations may affect the bound LPA activity to act as an intracellular messenger. Obviously, delineation of structure-activity relationship of LPA would largely help in understanding its precise biological function at molecular level. Generally, LPAR6-LPA binding activates Rho-signaling pathway which is a possible mediator of hair growth (Figure 5) [31]. Alternatively, LPAR6 may implicate in the regulation of hair growth through β-catenin dependent activity [32], [33]. Overall, a continued study of LPA in multiple independent pathways would assist in delineating additional clues for designing novel therapeutic targets to treat hair loss in humans.

## Supporting Information

Figure S1 **Ligplots showing comparative binding analysis of LPAR6^WT^ and LPAR6^mut^ interactions with LPA.**
(TIF)
