# Supplementary figures and images for: In Silico Analysis of Missense Mutations in LPAR6 Reveals Abnormal Phospholipid Signaling Pathway Leading to Hypotrichosis
Source: PLoS One. 2014 Aug 13;9(8):e104756. doi: 10.1371/journal.pone.0104756 (PMC4132050; doi:10.1371/journal.pone.0104756)

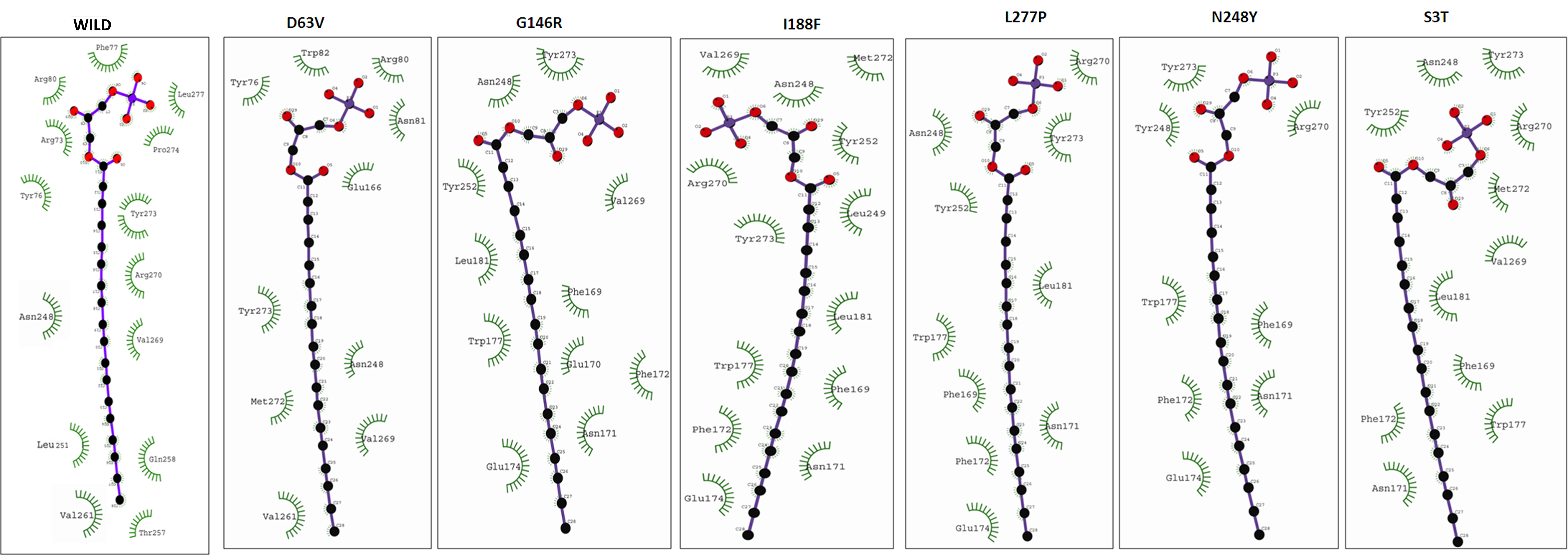

Supplement: Figure S1 — Ligplots showing comparative binding analysis of LPAR6WT and LPAR6mut interactions with LPA. (TIF) [file pone.0104756.s001.tif]
